# Biochemical and MALDI-TOF Mass Spectrometric Characterization of a Novel Native and Recombinant Cystine Knot Miniprotein from *Solanum tuberosum* subsp. *andigenum* cv. Churqueña

**DOI:** 10.3390/ijms19030678

**Published:** 2018-02-28

**Authors:** Juliana Cotabarren, Mariana Edith Tellechea, Sebastián Martín Tanco, Julia Lorenzo, Javier Garcia-Pardo, Francesc Xavier Avilés, Walter David Obregón

**Affiliations:** 1 Centro de Investigación de Proteínas Vegetales (CIPROVE), Departamento de Ciencias Biológicas, Facultad de Ciencias Exactas, Universidad Nacional de La Plata, 47 y 115 s/N, La Plata B1900AVW, Argentina; cotabarren.juliana@biol.unlp.edu.ar (J.C.); mariana.edith.tellechea@gmail.com (M.E.T.); 2 Institut de Biotecnologia i de Biomedicina, Universitat Autònoma de Barcelona, Campus Universitari, Bellaterra, Cerdanyola del Vallès, 08193 Barcelona, Spain; sebastiantanco@gmail.com (S.M.T.); julia.lorenzo@uab.es (J.L.); 3 Catalan Institute of Nanoscience and Nanotechnology (ICN2), CSIC and The Barcelona Institute of Science and Technology, Campus UAB, Bellaterra, 08193 Barcelona, Spain

**Keywords:** cystine-knot miniproteins, carboxypeptidase inhibitor, plant inhibitor, *Solanum tuberosum*, protease, Andean potatoes

## Abstract

Cystine-knot miniproteins (CKMPs) are an intriguing group of cysteine-rich molecules that combine the characteristics of proteins and peptides. Typically, CKMPs are fewer than 50 residues in length and share a characteristic knotted scaffold characterized by the presence of three intramolecular disulfide bonds that form the singular knotted structure. The knot scaffold confers on these proteins remarkable chemical, thermal, and proteolytic stability. Recently, CKMPs have emerged as a novel class of natural molecules with interesting pharmacological properties. In the present work, a novel cystine-knot metallocarboxypeptidase inhibitor (chuPCI) was isolated from tubers of *Solanum tuberosum*, subsp. *andigenum* cv. Churqueña. Our results demonstrated that chuPCI is a member of the A/B-type family of metallocarboxypeptidases inhibitors. chuPCI was expressed and characterized by a combination of biochemical and mass spectrometric techniques. Direct comparison of the MALDI-TOF mass spectra for the native and recombinant molecules allowed us to confirm the presence of four different forms of chuPCI in the tubers. The majority of such forms have a molecular weight of 4309 Da and contain a cyclized Gln in the N-terminus. The other three forms are derived from N-terminal and/or C-terminal proteolytic cleavages. Taken together, our results contribute to increase the current repertoire of natural CKMPs.

## 1. Introduction

Cystine-knot miniproteins (CKMPs or “knottins”) have recently emerged as a novel natural class of biomolecules with unique structural and functional properties [1,2]. CKMPs combine characteristics from proteins and peptides, displaying a protein-like fold but having a peptide-like size (>10 kDa) [1,2,3]. Typically, these molecules are 30–50 amino acids in length and share a common extremely stable tertiary fold, the so-called cystine-knot motif. This motif is formed of three antiparallel β-strands and connecting loops of varying length and amino acid composition. The whole knot structure (also termed the knottin scaffold) is held together by a characteristic disulfide-bridge connectivity composed of three disulfide bridges, which endows the resulting molecule with exceptional thermal, chemical, and proteolytic stability [1,2,4]. 

To date, a large number of CKMPs have been identified and isolated from natural sources (i.e., from arthropods, vertebrates, mollusks, fungi, plants, and so on). These molecules belong to more than 34 protein families and are involved in diverse biological functions [1,2]. In animals, CKMPs are secreted peptides that function as extracellular signaling molecules involved in the regulation of important cellular functions such as cell growth or cell development [5,6]. In plants, CKMPs are often involved in defense against external pathogens. Typically, the latter group functions as protease inhibitors and/or modulators of responses against pathogen attack [5,7,8]. Due to their extremely high stability, resistance to proteolysis, and interesting pharmacological properties, many natural CKMPs have gained much attention as novel peptide-based drugs for the treatment of a wide range of relevant human diseases such as chronic pain, infections, malaria, or ischemic strokes, among others [1]. One of the most prominent examples is the case of the calcium channel blocker ω-conotoxin MVIIa from the marine snail *Conus magus*. This cystine-knot miniprotein has recently been approved by FDA for the treatment of severe chronic pain [1,9]. Other relevant examples are the spider toxins Psalmopeotoxin I and II, which have been shown potent antiplasmodial activity against the malaria parasite *Plasmodium falciparum* [10,11]. Interestingly, several CKMPs isolated from plants (e.g., Pafp-S, circulin A, circulin B, cyclopsychotride, or kalata B1) have demonstrated antibacterial and/or antifungal activities [3,12,13,14].

A number of CKMPs function as potent protease peptide inhibitors (PPIs). Among them, the potato carboxypeptidase inhibitor (PCI) was the first CKMPs to be discovered [4]. In particular, PCI inhibits different enzymes within the family of metallocarboxypeptidases (MCPs), proteolitic enzymes that cleave C-terminal amino acids in proteins and peptides [15,16]. The first crystal structure of this 39 amino acid plant protease inhibitor was reported in 1980 by Rees and Liscomb [17]. After almost 40 years of intense research in the field of proteases, only a small number of metallocarboxypeptidase inhibitors (MCPIs) have been isolated and characterized so far [18,19,20,21,22,23,24,25,26,27,28,29]. Some of those novel PPIs are cystine-knot miniproteins isolated from species of the *Solanaceae* family of flowering plants, i.e., *Solanum tuberosum* (PCI), *Solanum lycopersicum* (MPCI) [18], *Capsicum annuum* (YBPCI) [29] and the variety of Andean potatoes *Solanum tuberosum*, subsp. *andigenum* cv. *Imilla morada* (imaPCI) [20]. In addition, other naturally occurring MCPIs without a knottin fold have been isolated from different animal species such as the intestinal parasite *Ascaris suum* (ACI) [30]; the medicinal leech *Hirudo medicinalis* (LCI) [23]; the tick *Rhipicephalus bursa* (TCI) [24] and *Hemaphysalis longicornis* (H1TCI) [25]; the marine mollusk *Nerita versicolor* (NvCI) [28], the marine ringworm *Sabellastarte magnifica* (SmCI) [31]; and rats and humans (i.e., latexin and its close homolog RARES-1). The use of natural PPIs to regulate MCPs’ action has emerged as a potential tool for the development of new therapeutic strategies. This is in agreement with the hypothesis that natural products will be among the most important sources of new bioactive drugs in the future [32,33]. In 2006 Wang and co-workers demonstrated that PCI can act as an antithrombotic drug [34]. Moreover, PCI was demonstrated to inhibit in vitro adenocarcinoma cell growth by acting as an epidermal growth factor (EGF) antagonist [35]. More recently, it has been demonstrated that the same inhibitor blocks the C-terminal cleavage (and consequent inactivation) of human EGF in vitro by the pancreatic carboxypeptidases A and B (CPA and CPB, respectively) [20]. 

Here we show the identification of a novel cystine-knot miniprotein, a member of the PCI-family present in *S. tuberosum* subsp. *andigenum* cv. Churqueña, a variety of potato cultivated along the Andean Cordillera in South America. This novel inhibitor, named chuPCI, was isolated from potato tubers, purified by affinity chromatography, and further characterized by matrix-assisted laser desorption/ionization time-of-flight (MALDI-TOF) mass spectrometry. The total RNA isolated from the tuber buds was employed for the cloning and expression of this novel inhibitor. The resultant recombinant product (rchuPCI) was purified until homogeneity and further characterized using mass spectrometry. Finally, the *Ki* values were determined against bovine CPA (bCPA) and porcine CPB (pCPB), two pancreatic MCPs. This work expands the current knowledge of CKMPs present in potatoes, one of the most cultivated and consumed crops all over the world. 

## 2. Results and Discussion

### 2.1. Identification and Initial Characterization of a Native Metallocarboxypeptidase Inhibitor from S. tuberosum subsp. andigenum cv. Churqueña

**Figure 1:**
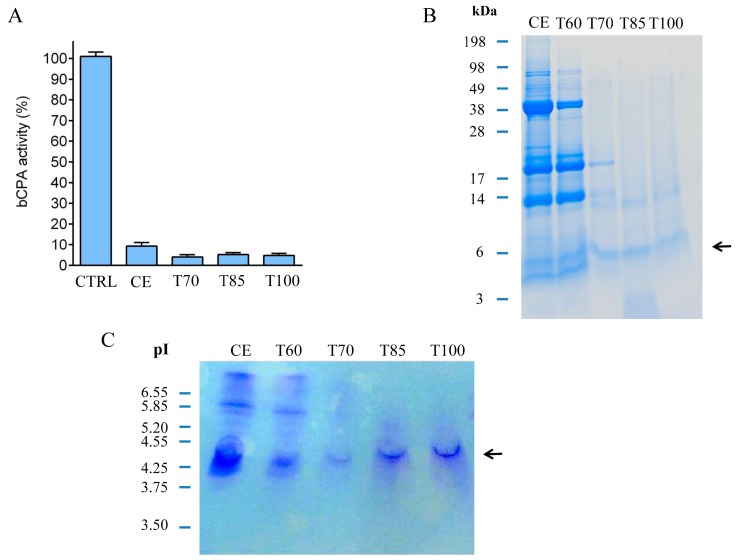
Thermal stability characterization of the Churqueña tubers crude extract. (**A**) Carboxypeptidase A (CPA) inhibitory activity of the non-treated crude extract (CE) and of the samples of the crude extract after incubation at (T70) 70, (T85) 85 and (T100) 100 °C for 60 min. The inhibitory activity of these samples is shown as % compared to (CTRL) a positive control condition of CPA without inhibitor; (**B**) Tris-tricine sodium dodecyl sulfate polyacrylamide gel electrophoresis (tris-tricine-SDS-PAGE) analysis of the crude extract (EC) and of the heat-treated samples after incubation at (T60) 60, (T70) 70, (T85) 85 and (T100) 100 °C for 60 min; (**C**) Isoelectrophocusing experiment of the crude extract (EC) and heat-treated samples incubated at (T60) 60, (T70) 70, (T85) 85 and (T100) 100 °C for 60 min. In panels (**B**,**C**) equal volumes (25 μL) of each sample were loaded in the gel, and the location of the potential *S. tuberosum* subsp. *andigenum* cv. Churqueña (native chuPCI) carboxypeptidase inhibitor is indicated with an arrow.

Members of the *Solanaceae* family of flowering plants are considered one of the most important sources of metallocarboxypeptidase inhibitors. In our study, we investigated the presence of carboxypeptidase inhibitors in an uncharacterized variety of potatoes; the *S. tuberosum* subsp. *andigenum* cv. Churqueña. This variety of Andean potatoes is endemic to the Andean Cordillera in South America, where it is extensively cultivated following local agro-ecological conditions. Several kilograms of fresh potato tubers were acquired from local growers. This material was used to prepare a crude extract achieved by crushing the tubers in distilled water. The resultant homogenate was incubated and centrifuged until we obtained a clear crude extract containing a large amount of the carboxypeptidase inhibitor. The total protein concentration of this sample was 790 μg·mL^−1^. The presence of the inhibitors in the final homogenate was confirmed by performing a dose–response inhibition experiment, as described previously [36]. The resultant IC_50_ value was 15.7 μg·mL^−1^. This IC_50_ value is comparable to those found in previous reports for other potato varieties [20,36]. Then, the clarified crude extract was subjected to thermal treatment at 60, 70, 85, and 100 °C for 60 min. In previous studies, it was demonstrated that MCPIs are heat-stable molecules due to the highly stable knottin scaffold [37]. After incubation at the indicated temperatures and centrifugation of the samples, the analysis of the soluble fraction using Bradford revealed an important reduction in the total protein content in all the heat-treated conditions (60 °C treatment = 112 μg·mL^−1^, 70 °C treatment = 88 μg·mL^−1^, 85 °C treatment = 92 μg·mL^−1^, 100 °C treatment = 82 μg·mL^−1^) in comparison with the soluble fraction of the untreated crude extract (CE = 789 μg·mL^−1^). The loss of protein content in the heat-treated samples can be explained by the denaturation of those heat-labile proteins. According to our results, these proteins represent more than 80% of the protein content when the crude extract was incubated at temperatures of 60 °C or higher. Simultaneously, the CPA inhibitory activity present in each of the samples of the crude extract and heat-treated conditions was determined (see Figure 1A). Results suggest the presence of a heat-stable CPA inhibitor in the crude extract. This molecule displayed high temperature stability, even after thermal treatment at 100 °C for 60 min (Figure 1A). Therefore, our results demonstrate that heat treatment at relatively high temperatures (up to 100 °C) mainly affects other proteins present in the crude extract, but not such miniproteins with a specific carboxypeptidase A inhibitory activity. Moreover, by heating the crude extract at temperatures higher than 60 °C we achieved a partial purification of the inhibitor. 

Proteins from each heat treatment were analyzed using tris-tricine sodium dodecyl sulfate polyacrylamide gel electrophoresis (tris-tricine-SDS-PAGE) and stained with Coomassie Blue after the electrophoresis. As shown in Figure 1B, the crude extract contains a variety of proteins of different molecular weight. The most abundant bands are those with an apparent molecular weight of around 40, 20, 13, and 4–5 kDa. Interestingly, thermal treatment at 70, 85, and 100 °C caused a notorious decrease in the intensity of those bands with a higher apparent molecular weight (>20 kDa). By contrast, bands of a lower apparent molecular weight displayed similar intensities after the aforementioned heat treatments (i.e., bands migrating at a molecular weight less than 14 kDa, and especially the band with an apparent molecular weight of 5 kDa). These results are in agreement with previous studies in which it was demonstrated that low molecular weight protease inhibitors are stable at high temperatures [23]. The isoelectric points (Ip) of all the proteins present in the crude extract and in the heat-treated samples were investigated by isoelectric focusing. The resultant isoelectrofocusing profiles showed the presence of a major band with an Ip of around 4.5 (Figure 1C). This Ip is comparable to what was previously described for potato carboxypeptidase inhibitors isolated from other potato varieties [20].

### 2.2. Purification and MALDI-TOF Mass Spectrometric Analysis of the Native Carboxypeptidase Inhibitor

**Figure 2:**
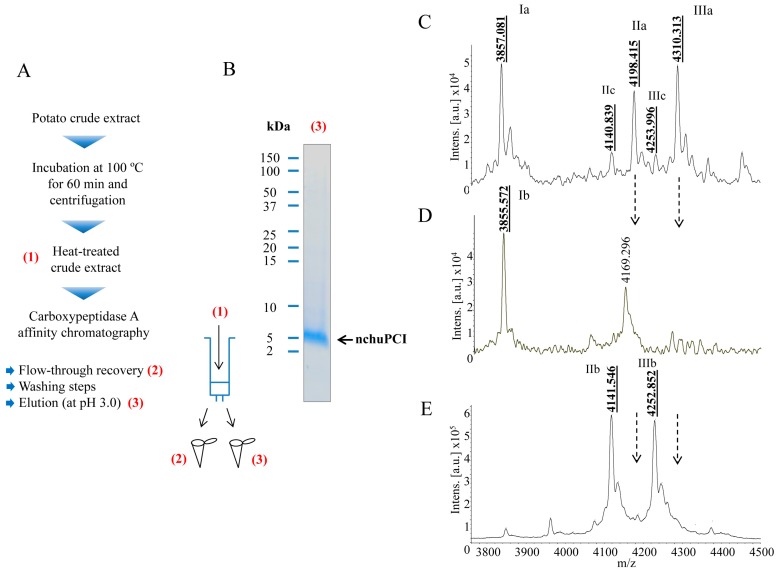
Purification and MALDI-TOF mass spectrometric analysis of a native carboxypeptidase inhibitor from Andean potatoes. (**A**) Schematic diagram of the one-step strategy followed for the purification of the native carboxypeptidase A inhibitor from potato tubers of *S. tuberosum* subsp. *andigenum* cv. Churqueña. Before purification, a crude extract of potato tubers was prepared and centrifuged. The clarified supernatant was incubated at 100 °C for 60 min. Once incubated, the sample was further centrifuged, and the resultant supernatant loaded onto an in-house prepared affinity resin for the specific purification of carboxypeptidase inhibitors; (**B**) Coomassie-stained tris-tricine-SDS-PAGE showing the purity of the final native protein. The location of the native chuPCI (nchuPCI) is indicated with an arrow; (**C**–**E**) MALDI-TOF mass spectra of (**C**) initial heat-treated crude extract, (**D**) unbound crude extract and (**E**) fraction eluted from the affinity chromatography. Numbers above the major peaks indicate the average mass of the MH+ ion (*m*/*z*). Note that the molecular mass is = mass of the MH+ −1 Da. MALDI-TOF spectra were recorded on a Ultraflextreme MALDI-TOF mass spectrometer (Bruker Daltonics) operating in in a linear positive mode. In panels D and E, dotted arrows indicate the mass of the MH+ ion (*m*/*z*) of the two faded signals (4198.415 and 4310.313 Da, corresponding to the peaks IIa and IIIa, respectively, in panel C).

MALDI-TOF mass spectrometric analysis of the crude extract and heat-treated samples (ut supra) revealed the presence in all the samples of one major peak corresponding to a miniprotein with a molecular mass of 4309 Da (Figure S1). This molecular weight mass is close to the expected molecular mass of the canonical potato carboxypeptidase inhibitor present in other varieties of potato tubers (4295 Da) [20,38]. As shown in Figure S1, this peak was observed practically unaltered in all the heat-treated samples (Figure S1). This result is in agreement with our previous results from inhibitory activity determinations and tris-tricine-SDS-PAGE analysis, and confirms that our novel miniprotein is a heat-resistant molecule. However, the size discrepancy observed between this particular molecule and the canonical PCI (16 Da) suggests that our miniprotein might be a distinct inhibitor or have a slightly different amino acid composition. To further study this miniprotein, we decided to purify this inhibitor from another batch of *S. tuberosum* subsp. *andigenum* cv. Churqueña potato tubers. For this purpose, purification of the inhibitor was achieved by performing a two-step purification protocol. As the first step, the extract was treated at 100 °C for 60 min for the removal of all the heat-labile proteins present in the sample. The second step was affinity chromatography (see Figure 2A and Materials and Methods Section 3.5.2 for details). For fast purification, we used an in-house fabricated resin by immobilizing bCPA in a glyoxyl agarose support in order to purify all the carboxypeptidase inhibitors from a fresh-prepared potato extract. This specific chromatography has been used in the past for fast purification of a wide range of carboxypeptidase inhibitors [37]. Protein elution was performed under acidic conditions (pH: ~3.0) in order to break the strong interactions that take place between bCPA and the putative binding molecules (see detailed purification protocol in Materials and methods, Section 3.5.2).

As shown in Figure 2B, after purification the resultant native miniprotein (hereafter termed native chuPCI) could be easily visualized on tris-tricine-SDS-PAGE by Coomassie blue staining, free of any major contaminating proteins. For further characterization, the initial heat-treated crude extract, the flow-through (fraction containing the non-retained peptides and proteins), and the elution from the affinity chromatography containing the final purified protein were subjected to MALDI-TOF mass spectrometry. A detailed analysis of the peaks present in the mass spectra for the initial heat-treated crude extract showed the presence of three major peaks (Figure 2C). The two most prominent signals showed a mass of 3857.1 Da (Ia) and 4310.3 Da (IIIa). The third majoritarian peak showed a mass of 4198.4 Da (IIa). In addition to these peaks, other secondary signals were observed with a molecular mass of 4140.8 Da (IIc) and 4254.0 Da (IIIc). When we analyzed the spectra of the flow-through, we observed a clear fading of the molecular ion intensities corresponding to the peaks with a mass of 4198.4 and 4310.3 Da (Figure 2C,D, peaks IIa and IIIa, respectively) as directly compared with the initial crude extract. However, in this fraction the peak at ~3856 Da stands out (Figure 2D, peak Ib), representing a peptide without interaction with the CPA immobilized support. To our surprise, in the fraction eluted from the affinity chromatography, two faded signals not visible in the crude extract appear in the mass spectra. These two peaks displayed a mass of 4141.5 and 4252.9 Da (see Figure 2E, peaks IIb and IIIb). In previous studies it has been demonstrated that the purification of carboxypeptidase inhibitors using this resin leads to the hydrolysis of the C-terminal amino acid present in the targeted inhibitor(s). In the particular case of PCI, a C-terminal glycine residue is usually cleaved by the immobilized CPA after the binding of the inhibitor [39,40]. This collateral cleavage causes the loss of 57 Da. This fits with the mass shift observed between the peaks IIa (4198.4 Da) and IIIa (4310.3 Da) and peaks IIb (4141.5 Da) and IIIb (4252.9 Da) from the affinity chromatography eluates. It is worth mentioning that the mass discrepancy between the two peaks in either the crude (IIa and IIIa) or eluates (IIb and IIIb) is 111 Da (Figure 2C,F). This mass difference fits well with the loss of an N-terminal residue such as glutamic acid or pyroglutamic acid from the miniprotein. The presence of the latter uncommon amino acid in the inhibitor can be explained by the potential post-translational cyclization of a Gln. Moreover, the presence of a peak with a mass of 4140.9 Da in the crude extract (Figure 2C, peak IIc), a mass similar to the molecular mass observed for IIIb (Figure 2F), suggests that a fraction of the inhibitor without the C-terminal Gly is present in potato tubers. 

According to our results, there is a mixture of different molecular species of the inhibitor in the potato tubers. Some of such species could be derived from post-translational proteolytic cleavage of either N-terminal or C-terminal amino acids. In addition, an important fraction of the native chuPCI is suspected to contain as N-terminal a pyroglutamic acid/glutamic acid residue. It is tempting to hypothesize that such post-translational modifications (i.e., proteolytic cleavages or the cyclization of Gln) might play a role in vivo. However, further studies will be needed to confirm this hypothesis.

### 2.3. Cloning and Sequence Analysis of the Novel Cystine-Knot Metallocarboxypeptidase Inhibitor (chuPCI)

**Figure 3:**
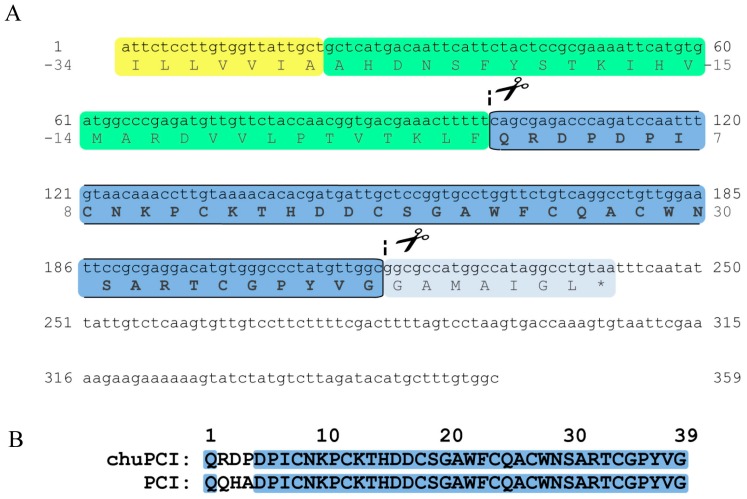
Nucleotide and deduced amino acid sequences of the chuPCI precursor. (**A**) cDNA of chuPCI was isolated from the RNA of tuber buds from *S. tuberosum* subsp. *andigenum* cv. Churqueña, a variety of Andean potato. The organization of the primary structure is indicated: residues belonging to the signal peptide (sequences highlighted in yellow); N-terminal pro-segment of the inhibitor (sequences highlighted in green); mature chuPCI (sequences highlighted in blue and boxed); C-terminal extension (sequences highlighted in grey). Scissors indicates the specific post-translational cleavage sites required to obtain the mature chuPCI molecule; (**B**) Sequence alignment of the deduced amino acid sequences of mature chuPCI and the sequence of canonical PCI (*S. tuberosum*, subsp. *tuberosum*, GenPept: NP_001275048.1). Conserved amino acids are highlighted in blue, while non-conserved residues are shown unmarked.

After exploring different sequencing methods, such as peptide mass fingerprint (PMF), Edman sequencing, or direct sequencing by LC-MS/MS, we did not obtain conclusive results for the sequence of the native chuPCI. For this reason, we decided to clone the inhibitor directly from the mRNA of the potato plant for its recombinant production. The total RNA content was isolated from tuber buds of *S. tuberosum* subsp. *andigenum* cv. Churqueña using a commercial kit. The RT-PCR yielded a 359 pb cDNA product. The PCR reaction employed to obtain the specific cDNA was performed with specially designed primers from conserved regions of *Solanaceae* family (see materials and methods Section 3.6.1 for details). The amplified cDNA was subjected to multiple sequence alignment using basic local alignment search tool (BLAST) [41]. This analysis revealed a high degree of similarity between chuPCI and sequences of members from the PCI family of inhibitors. According to the MEROPS database, these miniproteins are grouped as the I37 family of protease inhibitors [42]. Direct DNA sequencing of the resultant cDNA revealed that our sequence shares 96% of sequence identity with the canonical PCI (*S. tuberosum* metallocarboxypeptidase inhibitor IIa (PCI) precursor mRNA (GenBank: NM_001288119.1). The coding sequence of the cDNA (243 bp), which encodes the chuPCI precursor, has been deposited in the GenBank database (GenBank accession number MF682092). The nucleotide and deduced amino acid sequences of the chuPCI precursor are presented in Figure 3A.

As shown in the sequence alignment in Figure 3B, chuPCI conserves all the cysteine amino acids involved in the disulfide bond formation in the canonical PCI (Cys8–Cys24; Cys12–Cys27 and Cys18–Cys34). The same alignment also shows the location of those non-conserved residues between both inhibitors, which are located in the N-terminal region of the mature protein. Even though the C-terminal region is directly involved in carboxypeptidase inhibition mechanism, N-terminal modifications could produce structural modifications that change the stability towards diverse physicochemical parameters, such stability against temperature, pH, ionic strength, proteolytic digestion or differences on the inhibition kinetics. As mentioned above, the cystine-knot proteins are characterized by their compact and stable structure. To confirm that chuPCI has the knottin scaffold, the sequence of the mature miniprotein was analyzed with the knoter1D predictor from the KNOTTIN database webpage [43]. As expected, results confirmed that our novel inhibitor is cystine-knot miniprotein. 

### 2.4. Expression, Purification, and MALDI-TOF Mass Spectrometry Analysis of rchuPCI

**Figure 4:**
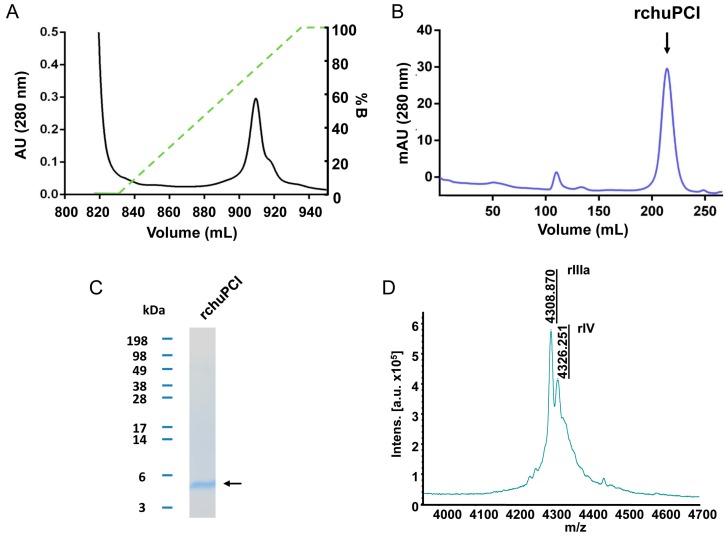
Expression and purification of the recombinant inhibitor (rchuPCI). rchuPCI was produced in *E. coli.* After 20 h expression at 37 °C, the recombinant miniprotein from the culture supernatant subjected to a disulfide reshuffling procedure. Then, the rchuPCI was purified from the clarified supernatant through two purification steps: a mixed-mode chromatography with a cation exchange ligand (Streamline Direct HST) and a gel filtration chromatography (HiLoad 26/60 Superdex 30 prep grade column). (**A**) Mixed-mode chromatography elution profile. Protein elution was carried out with an increasing gradient of phosphate buffer at pH = 8.5 (left *Y* axis). The green dashed line indicates the percentage of buffer B (100 mM sodium phosphate buffer at pH 8.5); (**B**) Size-exclusion chromatography elution profile of chuPCI. The peak corresponding to the elution of rchuPCI is inidicated (rchuPCI); (**C**) Tris-tricine-SDS-PAGE electrophoresis of the purified rchuPCI. The arrow indicates the location of the purified miniprotein; (**D**) MALDI-TOF mass spectrum of purified rchuPCI. Numbers above the major peaks indicate the average masses of the MH+ ion (*m*/*z*). Note that the molecular mass is = mass of the MH+ −1 Da.

We expressed and purified chuPCI. The production of such miniprotein in large amounts is an essential step to achieve its adequate biochemical characterization. For this reason, we cloned the sequence of chuPCI into the pIN-III expression vector with the OmpA3 signal peptide sequence located at the N-terminal end of the chuPCI sequence (see Materials and Methods Section 3.6.1 for more details about the cloning). Then, the vector was transformed in *E. coli* BL21 (DE3). The expression of chuPCI was induced by adding IPTG to the culture. After, 20 h of expression at 37 °C, the extracellular medium was collected and subjected to a disulfide reshuffling procedure in the presence of cysteine/cystine in order to ensure a proper folding of the protein. After reshuffling, the recombinant protein present in the clarified supernatant was purified as described previously for other related inhibitors [20]. Briefly, recombinant chuPCI (hereafter rchuPCI) was purified from the clarified supernatant by combining two different purification steps: (1) a mixed-mode Streamline Direct HST adsorbent containing a cationic ligand; and (2) size-exclusion chromatography (Figure 4). As shown in Figure 4A, elution of rchuPCI from the mixed-mode chromatography was carried out by applying an increasing gradient of pH = 8.5. The elution of the recombinant protein was observed when the pH of the elution buffer reached a pH of ~6.5. Immediately after its elution, all the fractions containing the miniprotein (as determined by SDS-PAGE and MALDI-TOF analyses) were adjusted concentrated and loaded onto a HiLoad 26/60 Superdex 30 prep grade column to obtain a high-purity product. As shown in Figure 4C, the purified rchuPCI showed an apparent electrophoretic mobility in agreement with its theoretical molecular weight (4325.8 Da).

MALDI-TOF mass spectrometric analysis of the purified rchuPCI was carried out. As observed in the mass spectra in Figure 4D, rchuPCI displays a two major peak with a molecular mass of 4308.9 Da and 4326.3 Da. The mass of the first peak is tightly close to the mass of the major peak present in the heat-treated crude extract of potato tubers (Figure 2C). The molecular mass of the latter peak fits with the theoretical molecular weight for rchuPCI of 4325.8 Da. The mass difference between both peaks is 17 kDa, which fits well with the cyclization of the N-terminal Gln. This confirms the presence of the N-terminal pyrogluamic acid in the native inhibitor and suggests that this post-translational modification is a common modification of the inhibitor that takes place in both plants and during the recombinant production of the inhibitor. One possible biological role for such modification could be protection against endogenous aminopeptidases, as demonstrated previously for other proteins and peptides [44,45]. 

### 2.5. Determination of the Inhibition Kinetic Constants (Ki) against A/B-Type MCPs

The inhibitory properties of the purified rchuPCI against bCPA and pCPB were determined and compared with a recombinant version of the canonical PCI. The *Ki* values were evaluated following a pre-steady-state based protocol, according to the Morrison model for tight-binding inhibitors. All *Ki* values determined for rchuPCI (summarized in Table 1) were found in the nanomolar range, a characteristic of such tight-binding inhibitors of MCPs. When compared to the canonical PCI, the *Ki* values obtained for rchuPCI against bCPA and pCPB were about 2-fold higher than the *Ki* values obtained for recombinant PCI (rPCI). These results suggest that N-terminal modifications in rchuPCI lead to an overall decrease in the inhibition potency against the tested MCPs when compared with PCI.

### 2.6. Identification and De Novo Sequencing of the Native chuPCI

**Figure 5:**
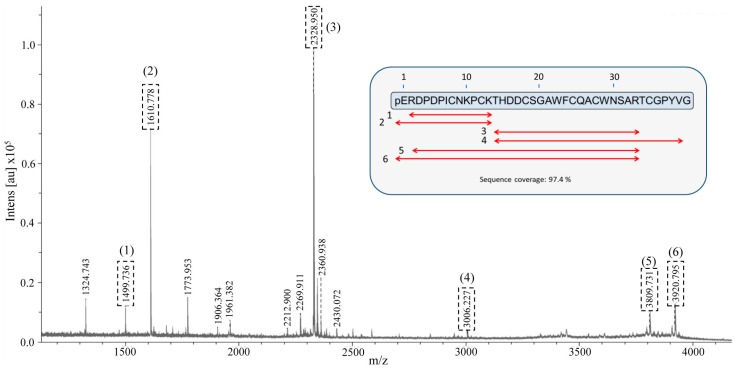
Peptide Mass Fingerprint profile of native chuPCI. MALDI-TOF mass spectra of the tryptic peptides resultant from the PMF analysis of the native chuPCI. Boxed peaks are those tryptic fragments that match the theoretical peptides generated by tryptic digestion of the rchuPCI sequence. For the analysis different post-translational modifications of rchuPCI were considered: (I) cyclization of the N-t Gln amino acid (conversion to pyroglutamic acid); (II) cleavage of the N-terminal Gln/cleavage of the N-terminal pyroglutamic acid; (III) cleavage of the C-terminal Gly. See Table S1 for more details. The inset shows the sequence coverage (red arrows) of each peptides from the PMF analysis. The final sequence coverage for the full-length inhibitor was 97.4% (100% for the C-terminally truncated inhibitor).

In the present work we have cloned and purified a novel inhibitor from a variety of Andean potatoes; *S. tuberosum* subsp. *andigenum* cv. Churqueña. By using MALDI-TOF mass spectrometry we found that the molecular mass of the recombinant inhibitor is similar to the molecular mass observed for one of the species of the native inhibitor present in potato tubers. However, we were not able to demonstrate whether our recombinant inhibitor corresponds to the native molecule identified in the tubers. To definitively confirm the sequence and identity of the native molecule we performed peptide mass fingerprint (PMF). In the past, this technique was successfully used for protein identification of different isoproteins with a high degree of similarity [46,47]. To perform this analysis, the electrophoretic band corresponding to native chuPCI eluted from the affinity chromatography (see tris-tricine-SDS-PAGE results in Figure 2B) was cut from the gel and digested with trypsin. Once extracted, tryptic peptides were analyzed by MALDI-TOF mass spectrometry. The corresponding mass spectrum for the resultant tryptic peptides is shown in Figure 5. An online MASCOT search (URL http://www.matrixscience.com) was performed for the identification of the tryptic maps against all the peptide and protein sequences present in the database. However no matches were found for any of the plant inhibitor or any other known sequences. Nevertheless, a manual search based on the rchuPCI sequence and considering different post-translational modifications (i.e., cyclization of the N-terminal Gln and/or cleavage of the N-terminal and/or C-terminal residues) showed sequence coverage of 100% (mass error < 50 ppm). As shown in the inset in Figure 5 and Table S1, six tryptic peptides matched with the theoretical peptides generated from the digestion of different native chuPCI. Interestingly, two of the observed tryptic peptides include the N-terminal pyroglutamic acid. This result allows us to unequivocally confirm the presence of this modification in the native chuPCI inhibitor.

Finally, two of the MH+ ions (MH+ ion = 2328.950 and MH+ ion = 1610.778) from the PMF spectrum were selected for its MALDI-TOF-TOF tandem mass spectrometric fragmentation. The fragmentation spectrum obtained for the precursor ion MH+ 2328.950 was compared with all the sequences present in the MASCOT database (considering the carbamidomethylation of all Cys). The result from this search confirmed the expected amino acid sequence for this peptide (THDDCSGAWFCQACWNSAR) (Figure S2). The MASCOT search for the fragmentation of the precursor ion MH+ 1610.778 did not produce results. For this reason, we carried out de novo sequencing of this peptide. The results of the sequencing are presented in Figure S3. The sequence deduced from the TOF/TOF analysis corresponds to the expected sequence for the peptide with an N-terminal pyroglutamic acid (pEQRDPDPICNKPCK). It is noteworthy to mention that these two peptides together add up 32 of the 39 chuPCI amino acids, which stands for 82% sequence coverage. This result strongly supports the presence of the pyroglutamic acid in the N-terminus of the inhibitor. In addition, this result demonstrates that our cloned inhibitor rchuPCI isolated from Churqueña tuber buds is the same inhibitor that we purified from the potato extracts (native chuPCI). Taking into account this information, we can now confirm the nature of all the signals observed in the MALDI-TOF mass spectra for the affinity-purified native chuPCI. As summarized in Table 2, the Churqueña potato extract contains mainly the full-length chuPCI post-translationally modified with an N-terminal pyroglutamic acid (Figure 2C and signal IIIa in Table 2). The second most important fraction of the inhibitor (according to its relative intensity in the mass spectrum) lacks the N-terminal amino acid (signal IIa in Table 2). This suggests the presence of endogenous active aminopeptidases in the potato tuber that cleaves this residue, and reinforces the previous hypothesis that the cyclization of the N-terminal Gln might contribute to protecting the miniprotein from N-terminal proteolytic degradation. The other two minoritarian chuPCI signals present in the extract correspond to the chuPCI with or without the N-terminal pyroglutamic acid residue and lacking the C-terminal Gly (see IIIc and IIc in Table 2).

## 3. Materials and Methods

### 3.1. Materials and Reagents

Carboxypeptidase A (CPA) from bovine pancreas, Carboxypeptidase B (CPB) from porcine pancreas, sodium chloride, tris (hydroxymethyl) aminomethane, sodium dodecyl sulphate (SDS), β-mercaptoethanol (βME), Coomassie Blue G-250, *N*,*N*,*N*′,*N*′-tetramethyl ethylene diamine (TEMED), pH range ampholytes and bovine serum albumin were purchased from Sigma-Aldrich (St. Louis, MO, USA). *N*-(4-methoxyphenylazoformyl)-phenylalanine-OH potassium salt and *N*-(4-methoxyphenylazoformyl)-arginine-OH potassium salt were acquired from Bachem (Bubendorf, Switzerland). *E. coli* BL21 (DE3) strain was purchased from Novagen (Madison, WI, USA). pGEM-T Easy vector was obtained from Promega Corporation (Madison, WI, USA). Primers were synthesized by Invitrogen Corporation (Carlsbad, CA, USA). DNA Taq polymerase was purchased from Biotools B&M Labs, S.A. (Madrid, Spain). Restriction enzymes and DNA ladders were obtained from Roche Applied Science (Penzberg, Germany). DNA T4-ligase was supplied by New England BioLabs (Hitchin, UK). Pre-stained broad range molecular mass standard was purchased from Invitrogen Corporation. Solvents and other common reagents were purchased from Sigma-Aldrich.

### 3.2. Crude Extract Preparation

Tubers of *S. tuberosum* L., subsp. *andigenum* cv. Churqueña were obtained from local growers in Jujuy, Buenos Aires, Argentina. Tubers were kept at −80 °C until use. For the crude extract preparation, approximately 60 g of tubers were thawed at room temperature, peeled and crushed using a blender after the addition of 170 mL of cooled distilled water. During all the steps, the mixture was kept at low temperature (4 °C) to avoid possible protein denaturation. After achieve a complete homogenization, starch and other insoluble material were removed by sequential centrifugation. After incubation for 40 min at 4 °C, the homogenate was centrifuged (1) for 60 min at 3000× *g* at 4 °C and (2) for 60 min at 12,000× *g* at 4 °C. Then the clarified supernatant was collected in Eppendorf tubes, centrifuged at 14,500× *g*, filtered through Orange Sci PES 0.2 μm filters and immediately frozen at −80 °C until analysis.

### 3.3. Total Protein Quantitation

Protein concentration was measured by the Bradford’s assay, as described [48]. Bovine serum albumin (BSA) was used as standard. Different standard solutions (0, 2, 5, 10, 15, 20 and 25 μg·mL^−1^) were prepared. 150 μL of each standard solution or test sample were mixed with 150 μL of the commercial Bradford reagent After 10 min incubation at room temperature the absorbance at 595 nm was measured (Tecan Infinite M200 PRO, Männedorf, Switzerland). The protein concentration was determined by interpolating the absorbance of each sample with the BSA calibration curve. 

### 3.4. Carboxypeptidase A Inhibition Measurements and IC_50_ Determination

CPA inhibitory activity was determined by using the substrate *N*-(4-methoxyphenylazoformyl)-Phe-OH in a 96-well microplate assay [36,49]. Briefly, to determine the inhibitory activity present in the crude extract, a fixed amount of bovine CPA (50 nM) was pre-incubated with different concentrations of the extract (ranging from 0 to 300 μg·mL^−1^) in 0.1 M Tris-HCl buffer (pH 7.5) containing 0.2 M NaCl. After pre-incubation for 15 min at 37 °C, the chromogenic substrate was added to each reaction mixture at a final concentration of 0.1 mM. Immediately, the absorbance at 340 nm was measured every minute for 15 min. The IC_50_ value was calculated from the different dose-response curve assays by fitting the data to the following equation, as previously described [36]:

(1) $$Y=\frac{100}{1+10^{\left(x-\mathrm{logIC}50\right)}},$$

where *x* is the log-transformed protein concentration tested, *Y* is the relative bCPA activity (% compared to control condition in the absence of inhibitor). IC_50_ is the extract concentration required to have 50% of bCPA inhibition. 

The bCPA inhibitory activity of the crude extract and the heat-treated samples was determined similarly as described above for the determination of the IC_50_ values. However, in this case a unique concentration of the sample was tested. bCPA inhibitory activity (%) was calculated by using the following equation:

(2) $$Y=\frac{(\frac{\Delta Abs340\mathrm{nm}}{\Delta t(\min)}{)}_{\mathrm{potato}\;\mathrm{extract}}}{(\frac{\Delta Abs340\mathrm{nm}}{\Delta t(\min)}{)}_{\mathrm{control}}}\times 100$$

The method for tight binding inhibitors was used for the *Ki* determination [50]. Inhibitory assays were performed at 25 °C under equilibrium conditions ([E_0_]/*Ki* ≤ 10) with a fixed concentration of bCPA or pCPB and substrate (0.1 mM *N*-(4-methoxyphenylazoformyl)-Phe-OH or 0.1 mM *N*-(4-methoxyphenylazoformyl)-Arg-OH, respectively) in Tris-HCl buffer (pH 7.5) containing 0.2 M NaCl. Samples were pre-incubated for 15 min at RT before the addition of substrate. Then, the decrease in absorbance was measured at 340 nm in a Victor3 (Perkin Elmer, Boston, MA, USA) plate reader. *Ki* values were calculated by direct fitting of the fractional activities (*v_i_*/*v*_0_) to the Morrison equation and assuming a competitive inhibition modality (Equations (3) and (4)).

(3) $$\frac{v_{i}}{v_{0}}=1-\frac{({\left[E\right]}_{T}+{\left[I\right]}_{T}+K_{i}^{app})-\sqrt{{\left({\left[E\right]}_{T}+{\left[I\right]}_{T}+K_{i}^{app}\right)}^{2}}-4{\left[E\right]}_{T}{\left[I\right]}_{T}}{2{\left[E\right]}_{T}}$$

(4) $$K_{i}=\frac{K_{i}^{app}}{([S_{0}/K_{M}])+1}$$

Definitions are; *v_i_*/*v*_0_, fractional activity; [E], the total active enzyme concentration; [I], is the inhibitor concentration; $K_{i}^{app}$, apparent inhibition constant; and *Ki*, true inhibition constant. 

### 3.5. Thermal Stability Experiments and nchuPCI Purification by Affinity Chromatography

#### 3.5.1. Thermal Stability Experiments

Aliquots with 1.0 mL of the clarified crude extract (see Section 3.2) where incubated at 60, 70, 85 or 100 °C for 60 min. After incubation the samples were centrifuged at 14,500× *g* for 90 min at 4 °C to eliminate all thermally denatured proteins. Afterwards, the total protein content and the inhibitory activity of the non-treated crude extract and heat-treated samples were determined. 

#### 3.5.2. Affinity Chromatography Purification

A 50 mL aliquot of the clarified crude extract (with a protein concentration of (200 μg·mL^−1^) was loaded onto a 2.0 mL bovine CPA-glyoxyl-agarose column prepared in house. The affinity resin was prepared in house as described [37]. Before the addition of the crude extract, the affinity resin was previously equilibrated with 10 column volumes (cv) 0.1 M Tris-HCl buffer (pH 7.2) containing 0.2 M NaCl. Then, the column was washed with 5 cv of equilibration buffer. The retained proteins were eluted with two different buffers: (1) HCl solution with pH = 5.0 (2 cv) and (2) mM HCl solution with pH = 3.0 (2 cv). The eluted fractions were adjusted immediately to pH 7.0 by adding NaOH. All the fractions that presented CPA inhibitory activity were pooled and stored at −80 °C until analysis.

### 3.6. Cloning and Expression of rchuPCI

#### 3.6.1. Cloning and Sequence Analysis of chuPCI

Total RNA from *S. tuberosum* L. subsp. *andigenum* cv. Churqueña tuber buds was extracted using the RNeasy Plant Mini Kit (Qiagen, Hannover, Germany). In order to synthesize first-strand cDNA, a reverse transcription reaction was done using RoR1poli-(dT)_15_ primer (5′-CCGGAATTCACTGCAGGGTACCCAATACGACTCACTATAGGGCTTTTTTTTTTTTTTTTT-3′) and M-MuLV enzyme (Fermentas, Waltham, MA, USA).

The PCR specific primers (Fwd: 5′-ATTCTCCTTGTGGTTATTGCTGC-3′ and Rev: 5′-GCCACAAAGCATGTATCTAAGAC-3′) were designed based on conserved regions between cDNAs encoding carboxypeptidase inhibitor precursors in *S. tuberosum* subsp. *tuberosum* L. (GenBank: NM_001288119.1) and *S. lycopersicum* L. (GenBank: NM_001247005.2). The resultant cDNA was cloned into the pGEM-T Easy vector (Promega). The DNA construct was named pGEM_chuPCIprecursor. The sequence construct was sequenced by automatic DNA sequencing (Macrogen Inc., Seoul, Korea) and the resultant sequence for the chuPCI cDNA was deposited in the GenBank database (accession code MF682092).

#### 3.6.2. Expression and Purification of rchuPCI

The nucleotide sequence corresponding to the mature chuCPI (39 amino acids) was cloned into the expression vector pIN-III-OmpA3. The sequence for the OmpaA3 signal peptide was cloned into the N-terminal region of the inhibitor. The OmpA3 sequence was included to promote the secretion of the protein to the periplasm of the bacteria in order to facilitate the proper folding of the miniprotein. The sequence of chuPCI was amplified by PCR and was inserted in the pIN-III-OmpA3 vector between the restriction sites for XbaI and SalI present in the expression vector. Previously, the N-terminal sequence (including the OmpA3 sequence) was introduced by PCR-mediated extension. The extension of the OmpA3 sequence was performed similarly, as described by Lufrano et al [20]. In our case the primers were: Fwd_1: 5′-CTACCGTAGCGCAGGCCCAGCGAGACCCGGATCC-3′, Fwd_2: 5′-GCTATCGCGATTGCAGTGGCACTGGCTGGTTTCGCTACCGTAGCGCAGGCC-3′, Fwd_3: 5′-tctagaTAACGAGGGCAAAAAATGAAAAAGACAGCTATCGCGATTGCAGTGGC-3′, Rev: 5′-GTCAGAATTCCTAGCCAACATAGGGCCCACATG-3′. The resultant pIN-III-OmpA3 construct was transformed into *E. coli* BL21 (DE3). 

Recombinant protein expression was carried out as previously described for other potato inhibitors [20]. In brief, 1 L culture of *E. coli* BL21 (DE3) transformed with the construct was grown at 37 °C in LB medium containing 100 μg·mL^−1^ ampicillin (Amp) until it reached an absorbance at 600 nm of about 0.6. The expression of rchuPCI was induced by adding 0.2 mM IPTG to the cell culture. After 20 h incubation at 37 °C, the extracellular medium was collected and subjected to a disulfide reshuffling procedure in the presence of a mixture of cysteine/cystine (final concentrations of 4 and 2 mM, respectively). This step is essential to ensure proper folding of the recombinant protein [20,51]. After reshuffling, the sample was clarified by overnight incubation at 4 °C and pH 4.0 and then, purified as described previously for other related inhibitors [20]. After reshuffling, the pH of the solution was adjusted to pH 8.5 and the sample was incubated for 4 h at 4 °C. 

After incubation, the rchuPCI was purified. Prior to purification, the pH of the medium was adjusted to pH = 4 with 1M citric acid. Then, the sample was incubated for overnight at 4 °C. After centrifugation at 12,000× *g* for 25 min, the clarified supernatant was filtered through 0.45 μm filters and applied onto a mixed-mode exchange column (Streamline Direct HST, GE Healthcare Biosciences, Uppsala, Sweden). The column was previously equilibrated with 100 mM sodium citrate buffer (pH 3.5). rchuPCI was eluted from the column by applying an increasing linear gradient of 100 mM sodium phosphate buffer (pH 8.5). The eluates containing the inhibitor (as determined by tris-tricine-SDS-PAGE and MALDI-TOF measurements) were pooled, concentrated and loaded onto a HiLoad 26/60 Superdex 30 prep grade column (GE Healthcare). The elution of rchuPCI was carried out with an isocratic gradient of 10 mM phosphate buffer (pH 8.5) at a flow rate of 2.5 mL min^−1^. Finally, all the eluted fractions containing rchuPCI were pooled and concentrated using an Amicon Ultra 3 kDa centrifugal device (Millipore, Darmstadt, Germany). The protein concentration was determined by measuring the absorbance at 280 nm and assuming a theoretical extinction coefficient of 12,865 M^−1^·cm^−1^ [52].

### 3.7. General Characterization of Methods

#### 3.7.1. Tris-Tricine-SDS-PAGE

Tris-tricine-SDS-PAGE was used to determine the purity of the protease inhibitor during the different purification steps. In addition, this technique was used to analyze the protein composition of the potato crude extracts and heat-treated samples. Briefly, samples were subjected to denaturing electrophoresis in tris-tricine gels composed of a stacking gel (4% T, 3% C), a spacer gel (10% T, 3% C) and a separating gel (16.5% T, 6% C), which is especially suitable to resolve peptide mixtures [53]. Samples were mixed with Novex^TM^ tricine SDS Sample buffer (2×) and were incubated for 5 min at 95 °C. The electrophoresis was performed in a Mini-Protean III dual slab cell (Bio-Rad, Hercules, CA, USA). The voltage was kept constant (30 V) until the samples completely left the stacking gel, and then the voltage was increased up to 90 V. The voltage was maintained constant until the end of the chromatography. Gels were stained with 0.2% Coomassie Brilliant Blue G-250 (Sigma-Aldrich, St. Louis, MO, USA) for overnight.

#### 3.7.2. Isoelectrofocusing (IEF)

IEF was used to determine the isoelectric point of the native chuPCI inhibitors present in the crude extract and heat-treated samples. To perform the analysis, all the samples were previously desalted. Desalting of samples was achieved by adding five volumes of acetone to the samples. After centrifugation at 16,000× *g*, the insoluble pellet was dissolved in a fixed volume of distilled water. Then, samples were loaded onto polyacrylamide gels (5%) containing broad pH range ampholytes (Pharmalyte 3–10, Pharmacia, Lisbon, Portugal). Isoelectric focusing was carried out in a Mini IEF Cell (Model 111, Bio-Rad) under constant voltage conditions in three different steps (step 1: 100 V for 30 min, step 2: 200 V for 15 min and step 3: 450 V for 60 min). Gels were then fixed and stained with a 0.2% Coomassie Brilliant Blue G-250 solution.

#### 3.7.3. MALDI-TOF Mass Spectrometric Analysis

For MALDI-TOF mass spectrometric analysis, samples from the different purification steps, as well as from the clarified crude extract were mixed with an equal volume of α-cyano-4-hydroxycinnamic acid (hcca) matrix solution. Previously, the samples were desalted by dialysis in 0.022 μm filters. The matrix-sample mixture was spotted onto MT 384 target plate polished steel T F from Bruker Daltonics, and the spots were evaporated to dryness at room temperature. Mass spectra were acquired on a Bruker Daltonics Ultraflextreme MALDI-TOF mass spectrometer (Bruker Daltonics, Billerica, MA, USA) operating in a linear positive mode. PMF analysis was performed for the identification of nchuPCI. In brief, an in situ tryptic digestion of the tris-tricine-SDS-PAGE electrophoresis bands as performed by following a well described protocol [46]. The resultant tryptic peptides were dissolved in 10 μL of 0.1% CF_3_CO_2_H (*v*/*v*) an analyzed by MALDI-TOF in the presence of hcca matrix. Mass spectra of the tryptic peptides were acquired in a reflectron positive mode with 25 kV acceleration voltage. In both cases, external calibrations were performed by using standard peptide calibration mixtures from Bruker Daltonics. Two precursor ions MH+ (1610.778 and 2328.950) were subjected to TOF-TOF fragmentation. The resultant fragmentation spectra for these precursor MH+ ions were compared with all the fragmentation patters for all the sequences present in the MASCOT database (considering the carbamidomethylation of all Cys). In addition, de novo sequencing of the fragmentation pattern for the ion MH+ 1610.778 was carried out using BioTools software (version 3.2, Bremen, Germany).

## 4. Conclusions

In the present study, a novel A/B-type metallocarboxypeptidase inhibitor from *S. tuberosum* subsp. *andigenum* cv. Churqueña (chuPCI) was cloned, expressed, purified, and thoroughly characterized. chuPCI has natural mutations in three N-terminal residues when compared with prototype molecule of the PCI-family of inhibitors (PCI from *S. tuberosum* subsp. *tuberosum*). Kinetic measurements for the recombinant inhibitor showed lower *Ki* values against both bCPA and pCPB in comparison with the canonical PCI. By using MALDI-TOF mass spectrometry and direct comparison between the native and recombinant forms, four species of the carboxypeptidase inhibitor were identified in the crude extract of the Churqueña tubers. The sequence of these molecular species was confirmed by PMF MALDI-TOF MS/MS and de novo sequencing. One of these variants corresponds to chuPCI with an N-terminal pyroglutamic acid. This little-studied modification results from the cyclization of first Gln residue. The second majoritarian signal corresponds to chuPCI without the pyroglutamic acid at the N-terminus, and the third and fourth forms correspond to chuPCI with or without the N-terminal pyroglutamic acid both lacking the C-terminal Gly.

It is important to remark that in this work we were able to clone a novel inhibitor present in a singular variety of Andean potatoes, and this recombinant protein was the same as is present in large amounts in the tubers. We combined two different approaches to demonstrate the presence of this novel inhibitor in the tubers: molecular biology and mass spectrometric analysis. The combination of these two strategies allowed us to unravel the complexity of chuPCI variants present in Churqueña potato tubers, thus contributing to increased knowledge about natural MCPIs. In addition, our work contributes to increase the repertoire of bioactive cystine-knot miniproteins isolated from natural sources, which are potentially useful for biomedical applications. Moreover, our thorough characterization of the native inhibitor using mass spectrometry allowed us to identify the presence of post-translational modifications (i.e., the cyclization of the N-terminal Gln and N- and C-terminal proteolysis) not detectable by other conventional techniques. 

## Figures and Tables

**Table 1:** Inhibition kinetic constants (*Ki*) of rPCI and recombinant churqueña PCI (rchuPCI) for bovine carboxypeptidases A (bCPA) and porcine carboxypeptidases B (pCPB).

Enzyme	rPCI	rchuPCI
	*Ki* (nM)	*Ki* (nM)
**bCPA ^a^**	4.3 ± 0.46	10.8 ± 1.55
**pCPB ^b^**	10.7 ± 1.17	19.8 ± 1.82

^a^ Measurements were carried using the synthetic substrate *N*-(4-methoxyphenylazoformyl)-Phe-OH; ^b^ Measurements were carried using the synthetic substrate *N*-(4-methoxyphenylazoformyl)-Arg-OH.

**Table 2:** Summary of the different chuPCI molecular species observed in the MALDI-TOF mass spectrometric analysis of the crude extract and purified rchuPCI.

Source	Signal/Peak	Sequence	Theor M	Obs M	Error (ppm)
CE	ND	QRDPDPICNKPCKTHDDCSGAWFCQACWNSARTCGPYVG	4325.830	-	-
CE	IIIa	pERDPDPICNKPCKTHDDCSGAWFCQACWNSARTCGPYVG	4308.799	4309.313	119
CE	IIIc	pERDPDPICNKPCKTHDDCSGAWFCQACWNSARTCGPYV-	4251.748	4252.996	293
CE	IIa	-RDPDPICNKPCKTHDDCSGAWFCQACWNSARTCGPYVG	4197.699	4197.415	−68
CE	IIc	-RDPDPICNKPCKTHDDCSGAWFCQACWNSARTCGPYV-	4140.647	4139.839	−195
RP	rIIIa	QRDPDPICNKPCKTHDDCSGAWFCQACWNSARTCGPYVG	4325.830	4325.251	97
RP	rIV	pERDPDPICNKPCKTHDDCSGAWFCQACWNSARTCGPYVG	4308.799	4307.870	−216

CE, MALDI-TOF mass spectrum of the heat-treated crude extract (see Figure 2C); RP, MALDI-TOF mass spectrum of purified recombinant chuPCI (see Figure 4D); ND, Not detected; Theor M, theoretical average mass; error (ppm) difference between Obs M and Theor M expressed in parts per million. Note that the observed mass corresponds to the mass of the corresponding MH+ ions +1 Da.

## Supplementary Materials

Supplementary materials can be found at http://www.mdpi.com/1422-0067/19/3/678/s1.

## Abbreviations

CKMPs	Cystine-knot miniproteins
MCPIs	Metallocarboxypeptidase inhibitors
chuPCI	Potato carboxypeptidase inhibitor isolated from churqueña variety
rchuPCI	Recombinant chuPCI
MALDI-TOF/MS	Matrix-assisted laser desorption/ionization-time of flight mass spectrometry
PMF	Peptide mass fingerprint
MCPs	Metallocarboxypeptidases
PPIs	Protease peptide inhibitors
PCI	Potato carboxypeptidase inhibitor
ACI	*Ascaris suum* carboxypeptidase inhibitor
LCI	*Hirudo medicinalis* carboxypeptidase inhibitor
TCI	Ticks carboxypeptidase inhibitor
H1TCI	*Hemaphysalis longicornis* carboxypeptidase inhibitor
NvCI	*Nerita versicolor* carboxypeptidase inhibitor
ECI	Endogenous carboxypeptidase inhibitor
SmCI	*Sabellastarte magnifica* carboxypeptidase inhibitor
CPA	Carboxypeptidase A
CPB	Carboxypeptidase B
SDS	Sodium dodecyl sulfate
βME	β-Mercaptoethanol
TEMED	*N*,*N*,*N*′,*N*′-Tetramethyl ethylene diamine
BSA	Bovine serum albumin
PCR	Polymerase chain reaction
