# Supplementary material for: Biochemical and MALDI-TOF Mass Spectrometric Characterization of a Novel Native and Recombinant Cystine Knot Miniprotein from Solanum tuberosum subsp. andigenum cv. Churqueña
Source: Int J Mol Sci. 2018 Feb 28;19(3):678. doi: 10.3390/ijms19030678 (PMC5877539; doi:10.3390/ijms19030678)
Supplement: Supplementary file 1 [file ijms-19-00678-s001.zip › ijms-253828_Supplementary material.pdf]

SUPPLEMENTARY INFORMATION

Biochemical and MALDI-TOF mass spectrometric characterization of a novel native and recombinant cystine knot miniprotein from *Solanum tuberosum* subsp. *andigenum* cv. Churqueña

Juliana Cotabarren [1, †], Mariana Edith Tellechea [1, 2, †], Sebastián Martín Tanco [2], Julia Lorenzo-Rivera [2], Javier Garcia-Pardo [3, *], Francesc Xavier Avilés [2, *] and Walter David Obregón [1, *]

[1] Centro de Investigación de Proteínas Vegetales (CIPROVE), Departamento de Ciencias Biológicas, Facultad de Ciencias Exactas, Universidad Nacional de La Plata, 47 y 115 s/N, B1900AVW, La Plata, Argentina; cotabarren.juliana@biol.unlp.edu.ar (J.C.), mariana.edith.tellechea@gmail.com (M.E.T.)
[2] Institut de Biotecnologia i de Biomedicina, Universitat Autònoma de Barcelona, Campus Universitari 08193, Bellaterra, Cerdanyola del Vallès, Barcelona, Spain; sebastiantanco@gmail.com (S.M.T.), Julia.Lorenzo@uab.cat (J.L.R.).
[3] Catalan Institute of Nanoscience and Nanotechnology (ICN2), CSIC and The Barcelona Institute of Science and Technology, Campus UAB, Bellaterra 08193, Barcelona, Spain.

* Correspondence: javiergarciapardo@msn.com (J.G.P), Tel.: +34-676-081557; FrancescXavier.Aviles@uab.cat (F.X.A.), Tel.: +34-606-873290; davidobregon@biol.unlp.edu.ar (W.D.O.), Tel.: +54-221-423-5333x57
† These authors contributed equally to this work.

Supplementary Tables

Table S1

Table S1 is submitted as an attached EXCEL file (Table S1.xlsx).

Supplementary Figures

Figure S1

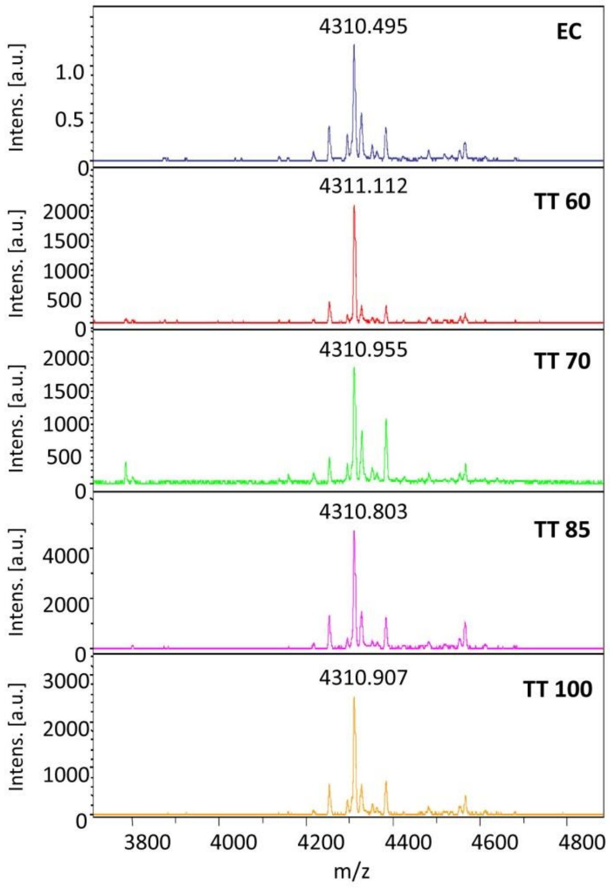

Figure S1. MALDI-TOF mass spectrometric analysis of the crude extract and crude extract thermal treatments. MALDI-TOF MS spectra of the non-treated crude extract (CE) and the same extract after incubation at (T60) 60, (T70) 70, (T85) 85 and (T100) 100 °C for 60 min. Numbers above the major peaks indicate the average masses of the MH+ ion (m/z). The peaks with a mass of about 4310 Da corresponds to a potential carboxypeptidase A inhibitor with a molecular mass of 4309 Da.

Figure S2

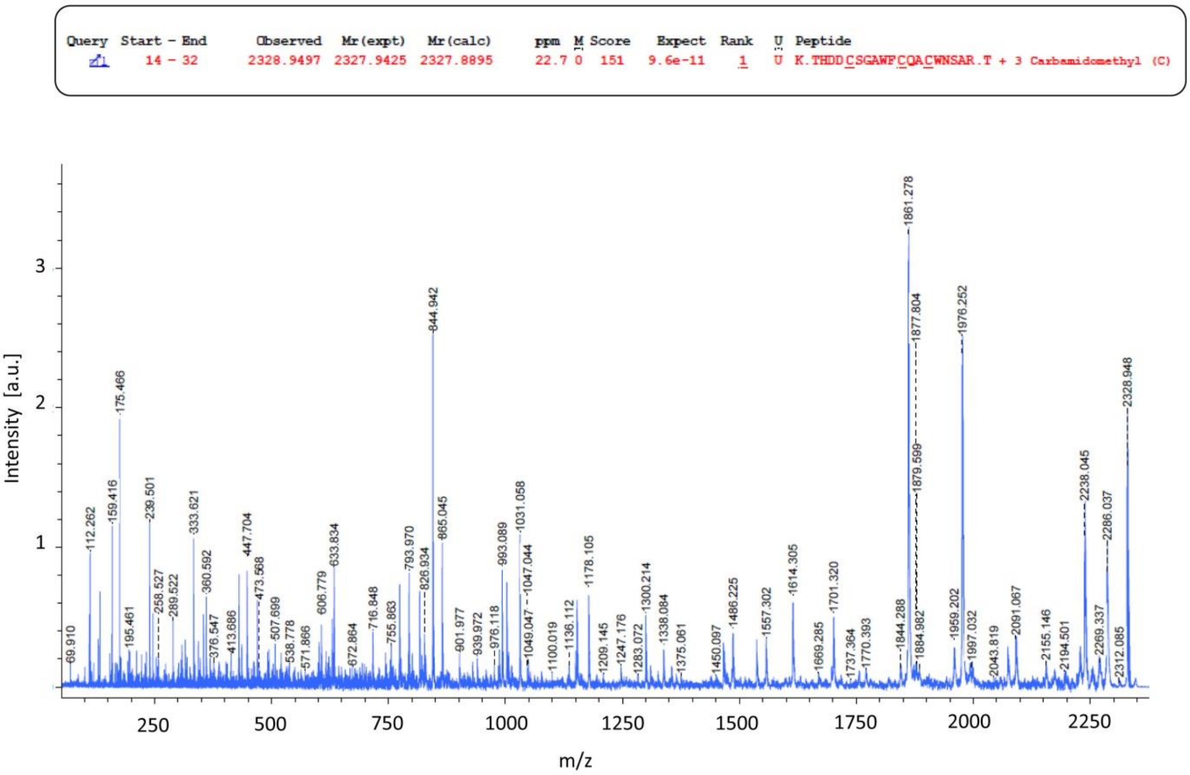

Figure S2. MALDI-TOF/TOF fragmentation spectrum of the precursor ion MH+ 2328.950. The inset above the spectrum shows the result obtained from the online MASCOT search.

Figure S3

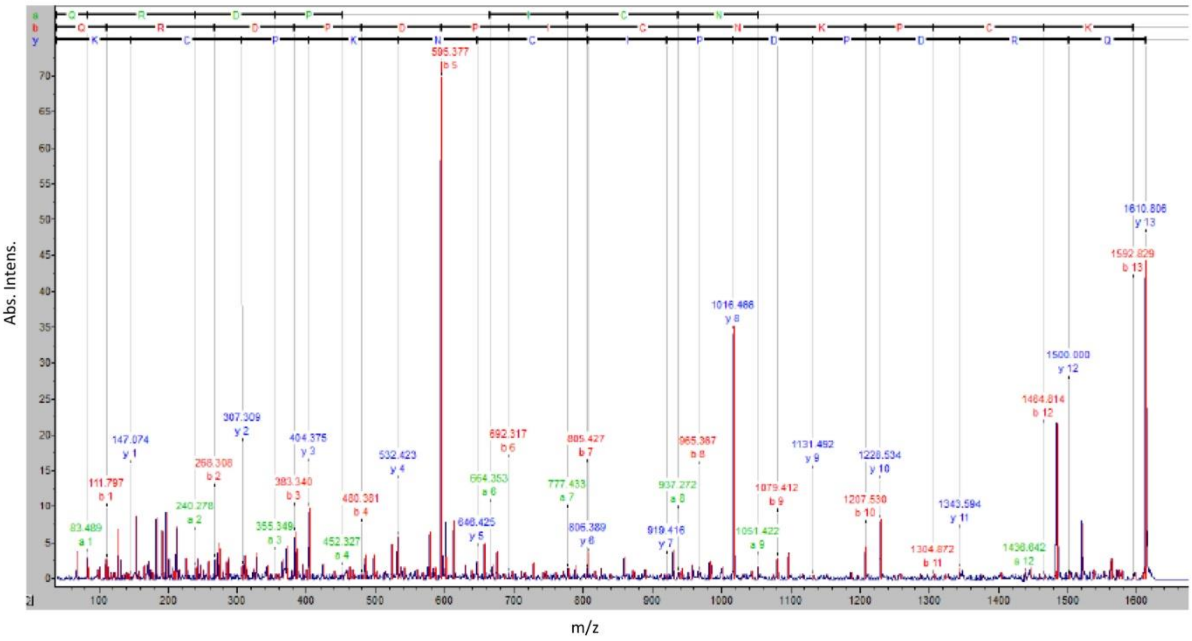

Figure S3. Representative *de novo* sequencing based on the MALDI-TOF/TOF fragmentation spectrum obtained for the precursor ion MH+ 1610.788.
